# Reducing excess antibiotic use with rapid tests to distinguish bacterial from nonbacterial causes of respiratory symptoms

**DOI:** 10.1017/ash.2026.10810

**Published:** 2026-09-01

**Authors:** Lao-Tzu Allan-Blitz, Jeffrey D. Klausner

**Affiliations:** 1 Division of Infectious Diseases, Department of Medicine, David Geffen School of Medicine, University of California Los Angeles, USA; 2 Department of Population and Public Health Sciences, Keck School of Medicine, University of Southern California, USA

Dear editor, we read with great interest the article by *Arensman Hannan et al.* which evaluated antibiotic prescribing practices among cases of acute sinusitis presenting to ambulatory care centers.^1^ The authors found that antibiotics were prescribed during more than 80% of encounters that did not meet prescribing criteria and that less than half of cases for which antibiotics were prescribed included doses and durations consistent with Infectious Disease Society of America and American Academy of Otolaryngology guidelines. Those findings highlight the immense burden of inappropriate antibiotic use among cases of sinusitis. Similar reports have been published for other diseases of the upper and lower respiratory tract.^2^

Pervasive overuse of antibiotics has been associated with numerous clinical and systems-level adverse outcomes. On a patient level, unnecessary antibiotic exposure increases the risk for adverse drug events, including allergic reactions, *C. difficile* colitis and other side effects.^3^ Disruptions to the microbiome attributable to antibiotic use have also been associated with increased risks for long-term metabolic disorders including obesity.^4^ On a systems level, inappropriate antibiotic prescribing practices lead to excess costs. Among children in the U.S., unnecessary antibiotic prescriptions were estimated to result in more than $24 million in attributable healthcare expenditures.^3^ Finally, on a population level, antibiotic use continues to drive and sustain the emergence of antimicrobial resistance—a public health crisis.

Novel strategies to support antimicrobial stewardship are urgently needed. One approach leverages serologic biomarkers that rule out bacterial infection. Myxovirus resistance protein A (MxA) is one such biomarker. MxA rises in response to type 1 interferon—which is induced specifically by viral infection and does not rise in bacterial infection.^5^ C-reactive protein (CRP) is an acute phase reactant that increases in response to a variety of stimuli including bacterial infection. A novel point-of-care, lateral flow test, FebriDx® (Lumos Diagnostics, United States), combines MxA and CRP detection. Across several trials the assay had a negative predictive value for bacterial infection near 99%.^6^ That test was recently FDA-cleared and Clinical Laboratory Improved Act-waived.

Results of combined MxA/CRP testing have been shown to directly impact clinical practice and antibiotic prescribing. One study evaluating the rapid MxA/CRP test among 256 children with symptoms of a respiratory tract infection found a 10% reduction in antibiotic use.^7^ A subsequent prospective study among 162 patients deemed likely to receive antibiotics for lower respiratory tract infections found that MxA/CRP testing led to a 41% reduction in antibiotic prescriptions. That study also followed patients for 28 days and found no increase in repeat visits among patients for whom antibiotics were deferred.^8^

Such tests contrast with molecular panels that aim to detect a wide array of pathogens. Pathogen-specific panels will always be limited by the number of microorganisms they detect, and frontline clinicians will not be able to defer antibiotics with confidence when no pathogen is detected. Indeed, a recent study found that antibiotic prescribing decreased by 50% when viral pathogens were detected on one such panel, but those changes were offset by increased antibiotic prescribing when no pathogen was detected. Use of the panel had no impact on clinical outcomes.^9^ Tests that measure host response, agnostic to specific pathogens, such as combined MxA/CRP testing, do not have the same limitations.

Use of combined MxA/CRP tests in ambulatory and acute care settings has the potential to transform antimicrobial stewardship efforts. Ambulatory settings that routinely observe excess or inappropriate antibiotic prescribing for respiratory illnesses are now able to rapidly categorize cases as nonbacterial. The potential benefits include increased physician confidence and patient comfort, lowered costs, and improved clinical outcomes. One cost model estimated that over 2.5 billion dollars would be saved annually in the U.S. by using a MxA/CRP assay with a 95% sensitivity for bacterial infections.^10^ Ambulatory centers described by *Arensman Hannan et al.* may benefit from consideration of MxA/CRP testing to avert inappropriate antibiotic use in patients.
